# Temperature-Responsive Lactic Acid-Based Nanoparticles by RAFT-Mediated Polymerization-Induced Self-Assembly in Water

**DOI:** 10.1021/acssuschemeng.3c01112

**Published:** 2023-06-26

**Authors:** Sarah E. Woods, James David Tinkler, Nabil Bensabeh, Marc Palà, Simon J. Martin, Ignacio Martin-Fabiani, Gerard Lligadas, Fiona L. Hatton

**Affiliations:** † Department of Materials, Loughborough University, Loughborough LE11 3TU, United Kingdom; ‡ Laboratory of Sustainable Polymers, Department of Analytical Chemistry and Organic Chemistry, University Rovira i Virgili, 43007 Tarragona, Spain

**Keywords:** RAFT, PISA, amphiphilic copolymers, green solvent, emulsion polymerization, biobased polymers, temperature-responsive, thermoresponsive

## Abstract

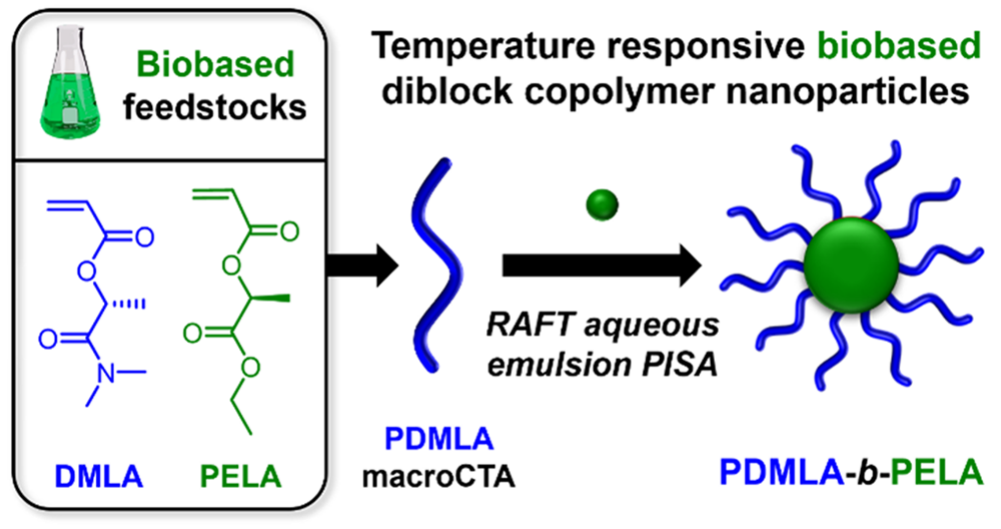

This work demonstrates for the first-time biobased, temperature-responsive diblock copolymer nanoparticles synthesized by reversible addition–fragmentation chain-transfer (RAFT) aqueous emulsion polymerization-induced self-assembly (PISA). Here, monomers derived from green solvents of the lactic acid portfolio, *N,N*-dimethyl lactamide acrylate (DMLA) and ethyl lactate acrylate (ELA), were used. First, DMLA was polymerized by RAFT aqueous solution polymerization to produce a hydrophilic PDMLA macromolecular chain transfer agent (macro-CTA), which was chain extended with ELA in water to form amphiphilic PDMLA-*b*-PELA diblock copolymer nanoparticles by RAFT aqueous emulsion polymerization. PDMLA_*x*_ homopolymers were synthesized targeting degrees of polymerization, DP_*x*_ from 25 to 400, with relatively narrow molecular weight dispersities (*Đ* < 1.30). The PDMLA_64_-*b*-PELA_*y*_ diblock copolymers (DP_*y*_ = 10–400) achieved dispersities, *Đ*, between 1.18 and 1.54 with two distinct glass transition temperatures (*T*_g_) identified by differential scanning calorimetry (DSC). *T*_g(1)_ (7.4 to 15.7 °C) representative of PELA and *T*_g(2)_ (69.1 to 79.7 °C) of PDMLA. Dynamic light scattering (DLS) studies gave particle z-average diameters between 11 and 74 nm (PDI = 0.04 to 0.20). Atomic force microscopy (AFM) showed evidence of spherical particles when dispersions were dried at ∼5 °C and film formation when dried at room temperature. Many of these polymers exhibited a reversible lower critical solution temperature (LCST) in water with a concomitant increase in *z*-average diameter for the PDMLA-*b*-PELA diblock copolymer nanoparticles.

## Abstract 2

Biobased diblock copolymer nanoparticles with temperature responsive properties were synthesized by RAFT aqueous emulsion PISA.

## Introduction

Environmental concerns surrounding fossil fuel-derived polymers have driven research into the polymerization of biobased monomers derived from renewable resources. Recent efforts have focused on biobased analogs of commodity plastics^1^ and polymerizing biobased monomers derived from renewable resources, including biomass^2^ and CO_2_.^3^ Advances in polymer science include the advent of reversible deactivation radical polymerization (RDRP) techniques which allow for the synthesis of well-defined polymers and the ability to access more complex copolymer compositions, such as block copolymers.^4^ RDRP of biobased monomers has recently become an area of interest,^5−7^ including synthesizing biobased block copolymers from various resources such as lignocellulosic biomass and vegetable oils.^7^ Block copolymers can self-assemble in bulk and solution when the blocks have dissimilar properties,^8,9^ making them desirable in applications like pressure-sensitive adhesives,^10^ thermoplastic elastomers (TPEs),^11^ and coatings.^12^

The well-known commodity chemical lactic acid can be accessed by microbial fermentation of carbohydrates from lignocellulosic biomass.^13^ While it is commonly associated with poly(lactic acid), it is an intermediate in the preparation of other valuable chemicals, including alkyl lactates and BASF’s Agnique AMD 3L (*N,N*-dimethyl lactamide, DML) solvent.^14^ Recently, Lligadas and co-workers developed a series of biobased monomers prepared from lactic acid derivatives, including ethyl lactate (EL). They demonstrated successful single-electron transfer-living radical polymerization (SET-LRP) forming homo- and block copolymers.^15^ Expanding on this work, amphiphilic block copolymers were synthesized based on ethyl lactate acrylate (ELA) and *N*,*N*-dimethyl lactamide acrylate (DMLA),^16^ as DMLA is water miscible and ELA immiscible with water. These amphiphilic block copolymers were investigated as surfactants used to stabilize monomer droplets in emulsion polymerization. Raffa et al. recently investigated the temperature responsive behavior of random- and block copolymers based on DMLA and ELA.^17^ While they noted that the PDMLA chemical structure, containing a substituted amide, is similar to other temperature responsive polymers, for example poly(*N*-isopropylacrylamide), a temperature response was only observed for PDMLA–PELA copolymers.

Polymerization-induced self-assembly (PISA) can be used to prepare block copolymer nanoparticles directly in water. Typically, a hydrophilic polymer is chain extended with a monomer in either a dispersion or emulsion polymerization, forming an amphiphilic block copolymer that self-assembles in situ.^18^ While PISA is often reported in combination with reversible addition–fragmentation chain transfer (RAFT) polymerization, other RDRP techniques have been used.^19^ RAFT polymerization^20^ allows for the synthesis of well-defined block copolymers,^21^ and it was recently highlighted as a promising technique to polymerize monomers derived from renewable resources.^22^ Moreover, the synthesis of stimulus-responsive block copolymer particles using PISA has been widely reported.^23,24^ Temperature is commonly used to elicit a change in polymer properties, for example, taking advantage of lower critical solution temperature (LCST) and upper critical solution temperature (UCST) behaviors, in aqueous conditions. This can result in changes to block copolymer particle morphology, such as a worm to sphere transition.^25^ However, there are no reports of biobased monomers in PISA formulations which are temperature-responsive.

Typically, reports of biobased monomers in RAFT-mediated PISA have not focused on generating fully renewably derived diblock copolymers.^26−29^ For example, Alexakis et al. demonstrated RAFT aqueous emulsion polymerization of the biobased terpene-derived monomer, sobrerol methacrylate, using a non-biobased macro-CTA.^29^ Naturally occurring biopolymers, such as polysaccharides, have been used as stabilizer blocks for RAFT-mediated PISA.^30−32^ This approach imparts a biobased block that is often chain extended with non-bioderived monomers, for example, Hatton et al. reported the synthesis of xyloglucan-stabilized poly(methyl methacrylate) (PMMA) latex particles to modify cellulosic reaction substrates.^31^ Coumes et al. chain extended poly(acrylic acid) (PAA) by RAFT dispersion polymerization with menthyl acrylate (MA), a monomer derived from menthol, in water/ethanol mixtures.^33^ The resultant PAA-*b*-PMA diblock copolymer nanoparticles can be fully biobased if the AA is synthesized from renewable resources (i.e., lactic acid). Recently, the same group chain extended PAA with lignin derivatives, acetoxy-protected 4-vinylguaiacol (AcVG), and *p*-hydroxystyrene (AcST) by RAFT emulsion polymerization forming PAA-*b*-PAcVG and PAA-*b*-PAcST diblock copolymer nanoparticles.^34^

**Figure 1:**
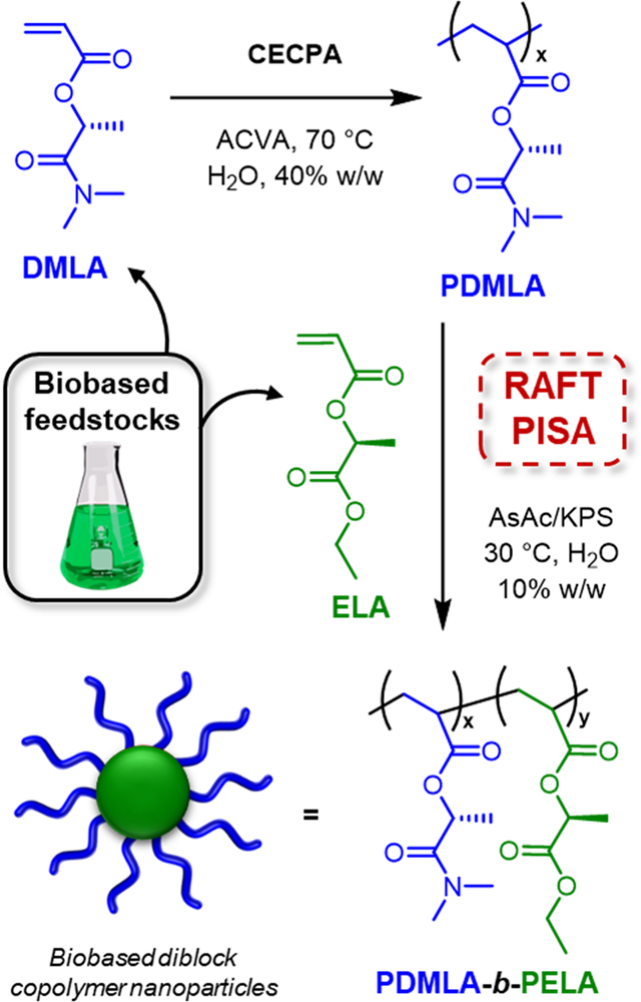
Schematic representation showing the synthetic approach to PDMLA_*x*_ macro-CTA by RAFT solution polymerization and subsequent chain extension with ELA forming PDMLA_*x*_-*b*-PELA_*y*_ diblock copolymer nanoparticles by RAFT (PISA).

Herein we report the synthesis of biobased diblock copolymer nanoparticles by RAFT aqueous emulsion PISA, using the renewable monomers DMLA and ELA, see Figure 1, synthesized from green solvents EL^35−37^ and DML.^38−40^ First, the RAFT aqueous solution polymerization of DMLA was optimized using various chain transfer agents (CTA) to form the PDMLA macro-CTA. Subsequently, a PDMLA_64_ macro-CTA was chain extended with ELA under RAFT aqueous emulsion polymerization conditions to form PDMLA_64_-*b*-PELA_*y*_ nanoparticles. Different reaction conditions were tested, and the self-assembled diblock copolymers were investigated for their solution properties and thermoresponsive behavior.

## Experimental Section

### Synthesis of PDMLA_*x*_ Using RAFT Solution Polymerization

For a typical reaction, when targeting DP_*x*_ of 50, DMLA (0.50 g, 2.92 mmol), 4-((((2- carboxyethyl)thio)carbonothioyl)thio)-4-cyanopentanoic acid (CECPA) (18.0 mg, 58.4 μmol), 4,4’-azobis (4-cyanovaleric acid) (ACVA) (3.28 mg, 11.7 μmol), and deionized water (40% w/w solids) were added to a reaction vessel. The unadjusted pH was measured (pH 3.4) using a Thermo Scientific Orion Star A211 benchtop pH meter before being sealed, degassed (N_2_) for 30 min, and submerged in an oil bath at 70 °C. After 1 h, the vessel was opened and left to cool to room temperature. A sample was removed for ^1^H NMR (D_2_O) to determine monomer conversion while the remaining polymer was purified using exhaustive dialysis against deionized water, using tubing with MWCO of 3.5 kDa for all homopolymers except the PDMLA_64_ where 1 kDa was used. After purification, the polymer was placed into a Thermo savant modulyo benchtop freeze-dryer overnight and characterized by ^1^H NMR (D_2_O) to determine the DP (DP_NMR_) and CTA efficiency (DP_*x*_/DP_NMR_), CHCl_3_ SEC for molecular weight data, and FTIR.

When alternative RAFT agents were used, the CECPA was replaced with 2-(2-carboxyethylsulfanylthiocarbonylsulfanyl)propionic acid (CPA) (14.9 mg, 58.4 μmol), 2-(dodecylthiocarbonothioylthio)-2-methyl propionic acid (DDMAT) (21.9 mg, 60.1 μmol), or 4-cyano-4-(phenylcarbonothioylthio)pentanoic acid (CPADB) (16.3 mg, 58.4 μmol), with DDMAT and CPADB in conducted dimethylsulfoxide (DMSO) instead of deionized water.

### Synthesis of PDMLA_64_-PELA_*y*_ Using Azo Initiator AIBA

For a typical reaction, when targeting a DP_*y*_ of 50, the PDMLA_64_ macro-CTA (0.14 g, 11.6 μmol), AIBA (12 μL of a 50 mg mL^–1^ stock solution, 2.32 μmol), ELA (0.10 g, 0.581 mmol), and deionized water (2.1 g) (10% w/w solids) were added to a reaction vessel. The reaction mixture was degassed (N_2_) for 30 min and submerged in an oil bath at 60 °C. After 2 h, the vessel was opened and left to cool to room temperature before the pH was recorded (pH 4.0). The diblock copolymer chains were analyzed by ^1^H NMR (DMSO-*d*_6_) to determine the monomer conversion, and a freeze-dried sample was analyzed by CHCl_3_ SEC for molecular weight data. The diblock copolymer nanoparticles were also characterized by DLS to establish particle z-average diameter, *D*_*z*_, and PDI.

### Synthesis of PDMLA_64_-PELA_*y*_ Using Redox Pair AsAc/KPS

A typical reaction, when targeting a DP_*y*_ of 50, started with three containers: PDMLA_64_ macro-CTA (0.27 g, 23.2 μmol) and KPS (26 μL of a 50 mg mL^–1^ stock solution, 4.65 μmol) in one, ascorbic acid (16 μL of a 50 mg mL^–1^ stock solution, 4.65 μmol) in the second, and ELA (0.20 g, 1.16 mmol) in the third. Deionized water (4.2 g) was split 80/20 between the first and second containers, and all three were degassed (N_2_) for 30 min. The ELA and ascorbic acid solution were transferred, respectively, into the first container before the contents were submerged in an oil bath at 30 °C. After 3 h, the vessel was opened and left to cool to room temperature before the pH was recorded (pH 2.8). The diblock copolymer chains and nanoparticles were characterized as described above for the azo-initiated polymerization.

## Results and Discussion

### RAFT Aqueous Solution Polymerization of DMLA

First, the RAFT aqueous solution polymerization of DMLA was investigated under varying conditions, targeting a DP_*x*_ of 50 with a CTA/initiator ratio of 5. In all polymerizations, ACVA was used as the initiator at a reaction temperature of 70 °C with 40% w/w solids content, while the CTA and solvent were varied. Four different CTAs were investigated, see Table 1 and Figure S1, including three trithiocarbonates and a dithiobenzoate: CECPA, CPA, DDMAT, and CPADB, respectively. Due to the poor aqueous solubilities of DDMAT and CPADB, these polymerizations were conducted in DMSO.

**Table 1:** Reaction Times, Monomer Conversions, Degree of Polymerization, CTA Efficiencies, and Molecular Weight Data Obtained for the Synthesis of PDMLA_50_ by RAFT Solution Polymerization at 70 °C, Using Four Different CTAs in Either H_2_O or DMSO

CTA	solvent	reaction time (h)	conversion^a^(%)	DP_*x*_ by NMR^a^	CTA efficiency^b^ (%)	*M*_n_^c^ (g mol^–1^)	*Đ*^c^
CECPA	H_2_O	17	99	52	96	5200	1.15
CPA	H_2_O	18	>99	65	77	7200	1.16
DDMAT	DMSO	17	99	57	87	7300	1.29
CPADB	DMSO	19	96	78	61	10,700	1.20

a Determined by ^1^H NMR analysis in D_2_O.

b Calculated using CTA efficiency (%) = DP_*x*_/DP_NMR_ × 100.

c Determined by SEC analysis using CHCl_3_ eluent containing 2% TEA and calibrated with a series of near-monodisperse PS standards.

Polymerizations were carried out for 17–19 h, and high conversions (≥96%) were obtained for all reaction conditions, as determined by ^1^H NMR analyses. When characterized using SEC, all PDMLA_50_ homopolymers had monomodal molecular weight distributions, with dispersities, *Đ*, between 1.15 and 1.29.

After purification by exhaustive dialysis, the DP from ^1^H NMR spectroscopy end-group analysis and CTA efficiency was calculated for each PDMLA_50_ homopolymer. See the Supporting Information for calculations, Figure S2–5 for representative ^1^H NMR spectra for CECPA, CPA, DDMAT, CPADB, and the corresponding purified PDMLA_50_ homopolymers, and Figure S6 for the FTIR characterization. As well as CECPA being water-soluble, it produced a PDMLA_50_ homopolymer with the lowest molecular weight dispersity and highest CTA efficiency. Thus, it was selected for use in subsequent RAFT aqueous solution polymerizations.

**Figure 2:**
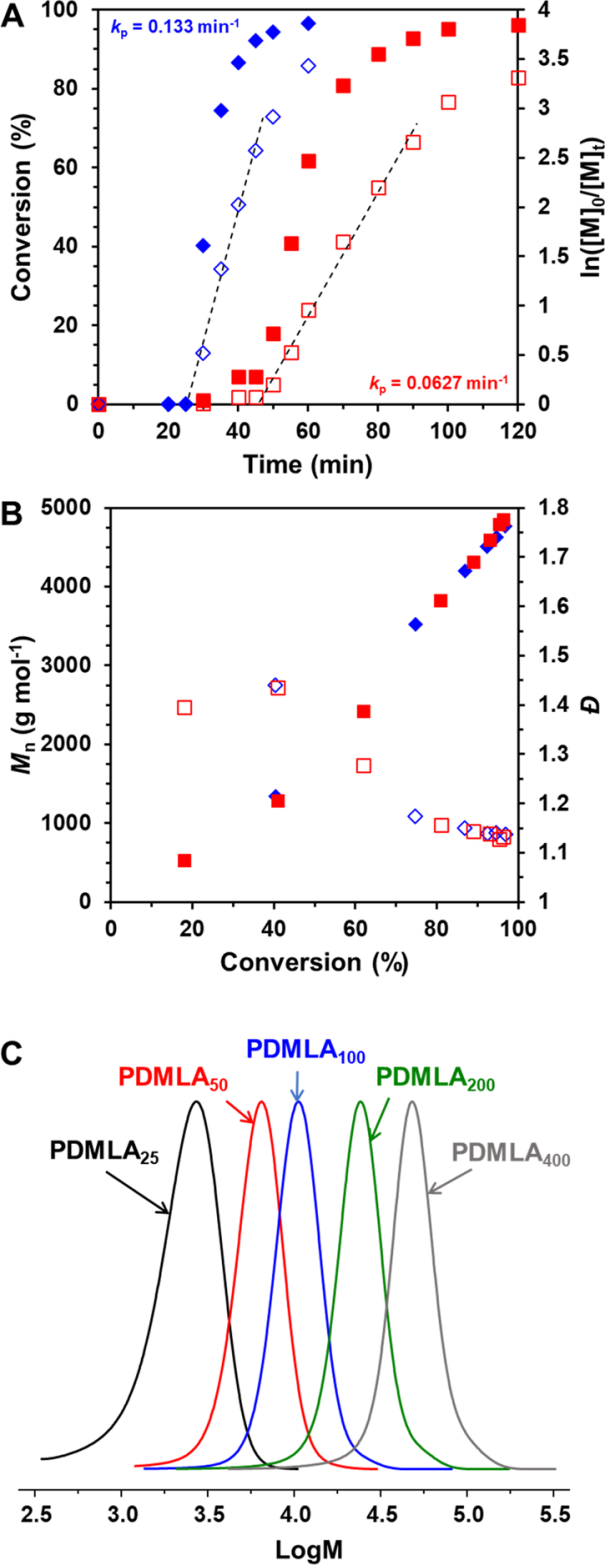
Kinetic plots for the RAFT aqueous solution polymerization of DMLA targeting a PDMLA DP of 50. (A) Conversion (filled symbols) and ln([M]_0_/[M]_t_) (open symbols) versus time with dashed lines representing linear fits to the data, and (B) *M*_n_ (filled symbols) and *Đ* (open symbols) versus conversion, with CECPA/ACVA ratios of 5 (blue diamonds) and 10 (red squares). (C) Overlaid normalized CHCl_3_ SEC chromatograms for PDMLA_*x*_ where *x* = 25, 50, 100, 200, and 400.

A kinetic study of the RAFT aqueous solution polymerization of DMLA was conducted (reaction conditions = [DMLA]_0_:[CECPA]_0_:[ACVA]_0_ = 50:1:0.2); see Figures 2A and B. The monomer conversion over time plot (Figure 2A) confirms a 25 min induction period followed by an increase in conversion to 97% in just 60 min, while the semilog plot demonstrates that the polymerization followed first-order kinetics concerning the monomer concentration, with a propagation rate coefficient, *k*_p_, of 0.133 min^–1^. Increasing the CECPA/initiator ratio from 5 to 10, whereby [DMLA]_0_:[CECPA]_0_:[ACVA]_0_ = 50:1:0.1, resulted in an extended induction period of nearly 45 min and a lower *k*_p_ (0.0627 min^–1^) with a monomer conversion of 96% in 120 min. SEC analyses (Figure 2B) showed a linear increase in *M*_n_ with increasing monomer conversion and a decrease in dispersity, *Đ*, in both cases. Although the CECPA/ACVA = 10 achieved a marginally lower dispersity (1.13 vs 1.14), to adhere to green chemistry principles,^41^ it was preferable to reach high conversion in a shorter time to minimize the energy used during polymerization.

Further PDMLA DPs of 25, 50, 100, 200, and 400 were targeted (Table 2) using CECPA/ACVA = 5, with all reactions reaching high monomer conversions of >96%. As demonstrated by SEC analyses, monomodal molecular weight distributions were observed (Figure 2C), low *Đ* were obtained (*Đ* < 1.3), and *M*_n_ increased linearly with increasing target DP, see Figure S7. DSC analyses determined the glass transition temperatures (*T*_g_) of the purified PDMLA_*x*_ homopolymers; see Table 2 and Figure S8. As expected, with increasing molecular weight, the *T*_g_ increased from 69.1 °C for PDMLA_25_ to 79.7 °C for PDMLA_400_.^42^

**Table 2:** Reaction Times, Monomer Conversions, Molecular Weight Data, and Glass Transition Temperatures Obtained for PDMLA_*x*_ Using RAFT Aqueous Solution Polymerization at 70 °C and a CECPA/ACVA Ratio of 5

target composition	reaction time (min)	conversion^a^(%)	*M*_n_^b^ (g mol^–1^)	*Đ*^b^	*T*_g_^c^ (°C)
PDMLA_25_	60	97	2000	1.28	69.1
PDMLA_50_	60	99	5700	1.14	74.0
PDMLA_64_	35	95	7900	1.15	77.0
PDMLA_100_	120	99	9800	1.12	78.1
PDMLA_200_	240	99	22,000	1.15	79.5
PDMLA_400_	360	98	44,600	1.15	79.7

a Determined by ^1^H NMR analysis in D_2_O.

b Determined by SEC analysis using CHCl_3_ eluent containing 2% TEA and calibrated with a series of near-monodisperse PS standards.

c Determined from the second heating cycle from DSC analyses of purified PDMLA_*x*_.

### RAFT Aqueous Emulsion Polymerization of ELA

To investigate the chain extension of PDMLA with ELA under RAFT aqueous emulsion conditions (Figure 1), first, a PDMLA macro-CTA was prepared, targeting a DP of 70. Previous work has shown that macro-CTA degree of polymerization can impact the particle morphology obtained by RAFT aqueous emulsion PISA. Hatton et al. demonstrated that while a poly(glycerol monomethacrylate) (PGMA) macro-CTA with a DP of 48 resulted in only spherical morphologies,^43^ shorter PGMA macro-CTAs (DP = 25, 28) allowed for the formation of non-spherical morphologies (i.e., worms/vesicles) for the RAFT aqueous emulsion polymerization of glycidyl methacrylate.^44,45^ Similarly, this was also demonstrated for the RAFT aqueous emulsion polymerization of 2-methoxyethyl methacrylate, monitored by small-angle X-ray scattering.^46^ Here, we targeted spherical morphologies, hence a longer macro-CTA chain length was synthesized. To ensure maximum chain-end fidelity, the reaction was stopped before full conversion was reached (at 35 min obtaining a 95% monomer conversion, see Table 2).^47^ The PDMLA macro-CTA was purified using exhaustive dialysis, the DP_NMR_ was calculated to be 64, and the SEC determined *M*_n_ was 7900 g mol^–1^ and *Đ* = 1.15.

### Investigation of Thermal Initiators

Initial studies into the RAFT aqueous emulsion polymerization of ELA were conducted using ACVA as the radical initiator at 70 °C, using a macro-CTA/initiator ratio of 5, targeting a PELA DP_*y*_ of 50 at 10% w/w solids with an unadjusted solution pH of 4.0. A high conversion (96%) was achieved after only a 2 h reaction time (Table S1). While the chain extension appeared successful, a high molecular weight tail was observed by analyzing the diblock copolymer chains by SEC (see Figure S9). This suggests either a loss of RAFT control or that branching due to chain transfer occurred, which has been observed for other RAFT PISA syntheses using acrylates.^48,49^ Moreover, the z-average diameter, *D*_*z*_, and polydispersity index (PDI) of the resulting diblock copolymer nanoparticles, determined by DLS analysis, were both higher than expected for discrete self-assembled diblock copolymer spherical particles (*D*_*z*_ = 69 nm, PDI = 0.28). Also, during this investigation, solids were observed during polymerizations, which became soluble upon cooling to room temperature. With further investigation, some PDMLA homopolymers and PDMLA-*b*-PELA copolymers exhibited temperature-responsive behavior (vide infra). Therefore, a second radical initiator was investigated, 2,2′-azobis-2-methyl-propanimidamide dihydrochloride (AIBA), as the 10 h half-life, *t*_10_, for this initiator is 58 °C, lower than ACVA (69 °C), allowing for the polymerizations to be conducted at 60 °C rather than 70 °C.

RAFT aqueous emulsion polymerizations of ELA were subsequently conducted using AIBA (macro-CTA/Initiator = 5) at 60 °C (Table S1). High conversions were obtained within 2 h reaction time (>97%). Targeting PELA DPs of 10, 25, and 50 resulted in PDMLA_64_*-b-*PELA_*y*_ diblock copolymers with monomodal molecular weight distributions and low dispersities (*Đ* = 1.18–1.32), see Figure S10. When targeting a higher core-forming block DP of 100, the molecular weight distribution became broad with a high molecular weight tail and a dispersity of 3.31, recorded by SEC analysis. This is likely due to both undesired termination events, which can occur in RAFT-mediated emulsion polymerization,^50^ and the propensity of acrylates to undergo excessive chain transfer, which can lead to branching and increased dispersity.^48−51^ As previously discussed, this was observed with both thermal initiators investigated, ACVA and AIBA, at 70 and 60 °C, respectively; others have observed that the amount of branching increases with temperature.^52^ Therefore, subsequent polymerizations were investigated at lower reaction temperatures.

### Varying the Solids Content

To investigate the effect of varying the solids content, the RAFT aqueous emulsion polymerization of ELA using AIBA (macro-CTA/Initiator = 5) at 60 °C was also conducted using a higher solids content of 20% w/w (see Table S1). Low dispersities were obtained for PDMLA_64_*-b-*PELA_10_ and PDMLA_64_*-b-*PELA_25_ diblock copolymers synthesized at 20% w/w, *Đ* = 1.18 and 1.22, respectively, with a slightly higher dispersity for PDMLA_64_*-b-*PELA_50_ (*Đ* = 1.39). Moreover, high conversions were observed within 2 h (>97%). Furthermore, the PDMLA_64_-*b*-PELA_*y*_ diblock copolymer nanoparticles synthesized at 10 and 20% w/w had similar z-average diameters, as determined by DLS analyses, see Table S1. Thus, the increase of solids content from 10 to 20% w/w for the polymerizations did not significantly alter the resulting block copolymer properties. As such, a solids content of 10% w/w was selected for further syntheses.

### Investigation of AsAc/KPS Redox Pair-Initiator

**Figure 3:**
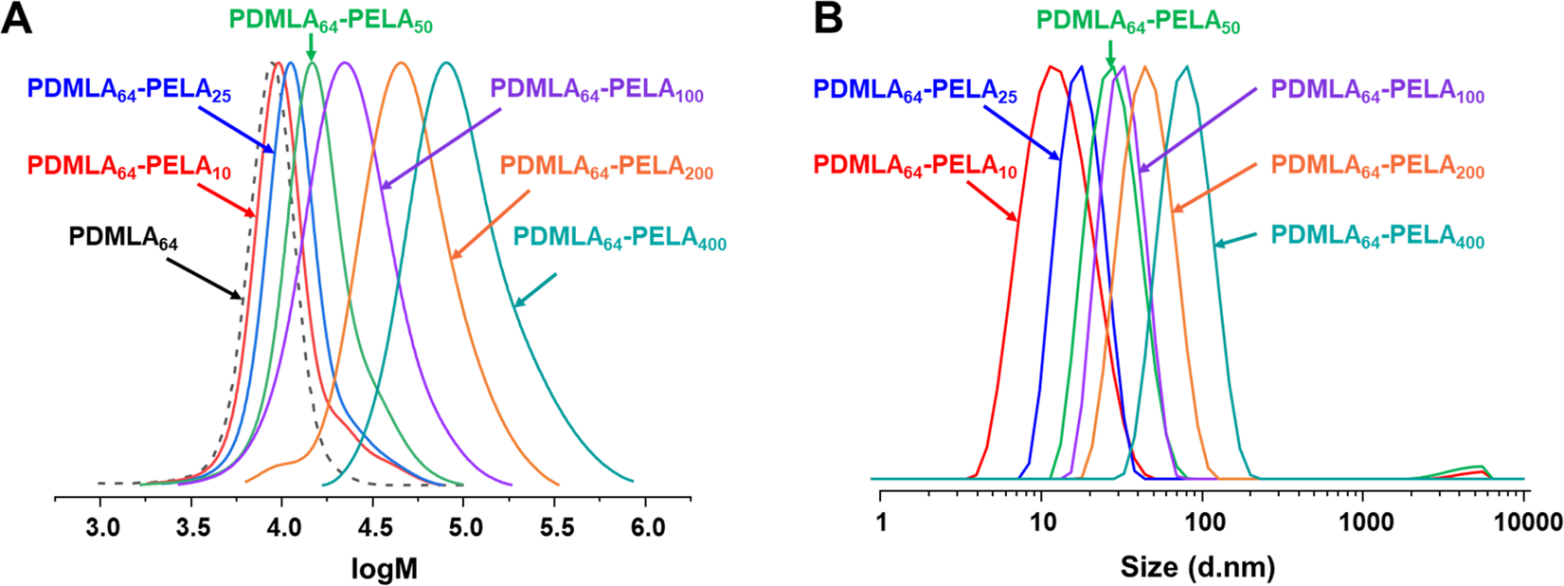
Results for PDMLA_64_-*b*-PELA_*y*_ diblock copolymers, where *y* = 10, 25, 50, 100, 200, 400, synthesized by RAFT aqueous emulsion polymerization of ELA using the redox pair AsAc/KPS at 30 °C. (A) Overlaid normalized SEC chromatograms and (B) DLS size distribution by intensity curves for nanoparticles.

Next, an ascorbic acid/potassium persulfate (AsAc/KPS) redox pair initiator system was investigated at a reduced reaction temperature of 30 °C, further reducing the energy required for these polymerizations, therefore improving the polymerizations “green” credentials.^41^ PDMLA_64_*-b-*PELA_*y*_ diblock copolymers, where *y* = 10, 25, 50, 100, 200, and 400, were targeted, maintaining a macro-CTA/initiator ratio of 5, at 10% w/w solids with a solution pH from 2.7 to 3.3, see Table 3. Under these mild RAFT aqueous emulsion polymerization conditions, high monomer conversions were achieved in 3 h reaction time, as determined by ^1^H NMR analyses. Loss of the vinyl protons from the ELA double bond between 5.92 and 6.47 ppm was observed over time, and the PDMLA_64_-*b*-PELA_*y*_ chemical structure was confirmed by ^1^H NMR (Figure S11). Characterization by SEC revealed an increase in *M*_n_ with increasing PELA core-forming block DP, with the dispersities, *Đ*, between 1.25 and 1.54. Chain extension of the PDMLA_64_ macro-CTA was evident from the shift in the molar mass distributions by SEC analyses (Figure 3A). Dispersities, *Đ*, increased with increasing core-forming block DP, which has also been observed in other RAFT aqueous emulsion polymerizations.^43,44,48^

**Table 3:** Monomer Conversions and Molecular Weight Data for the RAFT Aqueous Emulsion Polymerization of ELA, Using AsAc/KPS at 30 °C and 10% w/w Solids Content, Including Glass Transition Temperatures and DLS Data for the PDMLA_64_-*b*-PELA_*y*_ Diblock Copolymer Nanoparticles

target composition	conversion^a^ (%)	*M*_n_^b^ (g mol^–1^)	*Đ*^b^	*D*_*z*_^c^ (nm)	PDI^c^	*T*_g(1)_^d^ (°C)	*T*_g(2)_^d^(°C)
PDMLA_64_-*b*-PELA_10_	84	9400	1.27	11	0.18	–e	69.2
PDMLA_64_-*b*-PELA_25_	>99	10,900	1.25	17	0.08	–e	64.5
PDMLA_64_-*b*-PELA_50_	>99	14,100	1.30	27	0.20	15.7	55.0
PDMLA_64_-*b*-PELA_100_	97	19,300	1.47	30	0.07	10.8	63.8
PDMLA_64_-*b*-PELA_200_	>99	38,700	1.55	42	0.10	8.5	–e
PDMLA_64_-*b*-PELA_400_	>99	79,900	1.54	74	0.10	7.4	–e

a Determined by ^1^H NMR analysis in DMSO-*d*_6_.

b Determined by SEC analysis using CHCl_3_ eluent containing 2% TEA and calibrated with a series of near-monodisperse poly(styrene) standards.

c Determined by DLS at 5 mg mL^–1^.

d Determined from the second heating cycle using DSC.

e No *T*_g_ could be distinguished.

The PDMLA_64_-PELA_*y*_ diblock copolymer nanoparticles, where *y* = 10–400, were analyzed by DLS; see Table 3 and Figure 3B. The z-average diameters, *D*_*z*_, increased from 11 to 74 nm when increasing the PELA core-forming block DP from 10 to 400. By visual inspection, the PDMLA_64_-PELA_*y*_ diblock copolymer nanoparticle dispersions also became increasingly turbid with increasing PELA DP, while low polydispersity values were recorded for most of the PDMLA_64_-PELA_*y*_ diblock copolymer nanoparticles (PDI ≤ 0.10). The higher PDI values obtained for PDMLA_64_-*b*-PELA_10_ and PDMLA_64_-*b*-PELA_50_ diblock copolymer nanoparticles (0.18 and 0.20, respectively) can be attributed to the presence of a small peak between 1000 and 10,000 nm (Figure 3B) and may be due to a small degree of aggregation or presence of large contaminants.

The mean average diameter, *D*, of the nanoparticles is proportional to the core-forming block DP, y, as expressed by eq 1:
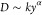
1where α is a scaling factor and *k* a constant.^53−55^ The relationship between the z-average diameters of the PDMLA_64_-*b*-PELA_*y*_ diblock copolymer nanoparticles and the PELA DP was found to fit a power law (Figure S12), giving an α value of 0.49. This parameter can be used to describe the behavior of the PELA core-forming chains, whereby a value close to 0.5, as we observe here, indicates unperturbed chain statistics and weak segregation with minimal solvation of the PELA chains in the core.^53−55^

The thermal properties of PDMLA_64_-*b*-PELA_*y*_ diblock copolymers, with DP_*y*_ = 10–400, were investigated using DSC analysis (Figure S13). Two glass transitions, *T*_g(1)_ and *T*_g(2)_, were observed for *y* = 50–400, suggesting phase separation of the two blocks. *T*_g(2)_ was identified in all of the copolymers between 55.0 and 69.2 °C, which was attributed to the PDMLA block. The PDMLA_64_ macro-CTA *T*_g_ was found to be 77.0 °C, and other PDMLA_*x*_ homopolymers exhibited glass transitions between 69.1 and 79.7 °C (see Table 1). The lower temperature transition, *T*_g(1)_, between 7.4 and 15.7 °C, for PDMLA_64_-*b*-PELA_*y*_ where *y* = 50–400 was associated with the hydrophobic PELA core-forming block as Bensabeh et al. previously reported the *T*_g_ of homopolymer PELA to be between −4 and 2 °C.^15,16^ The increased PELA *T*_g(1)_ and decreased PDMLA *T*_g(2)_ in the diblock copolymers, when compared with values for the corresponding homopolymers, suggest that the PELA is plasticizing the PDMLA to some extent, indicating some miscibility of the two blocks. This is corroborated by the relatively low value of α of 0.49, indicating weak segregation, as previously discussed. In contrast to previous findings, we observed a decrease in *T*_g(1)_ with increasing hydrophobic PELA core-forming DP. This suggests that increasing the PELA content in the diblock copolymers decreases the miscibility between the two blocks. Therefore, lower molecular weight PELA chains are more miscible with the PDMLA block than higher molecular weight chains, resulting in an increased *T*_g(1)_. It is well-known that molecular weight influences miscibility due to combinatorial entropy contributions when considering the thermodynamics of mixing.^56^ However, it is evident that the two blocks (PELA and PDMLA) are not fully miscible as a single *T*_g_ would be observed if this were the case.^57^

**Figure 4:**
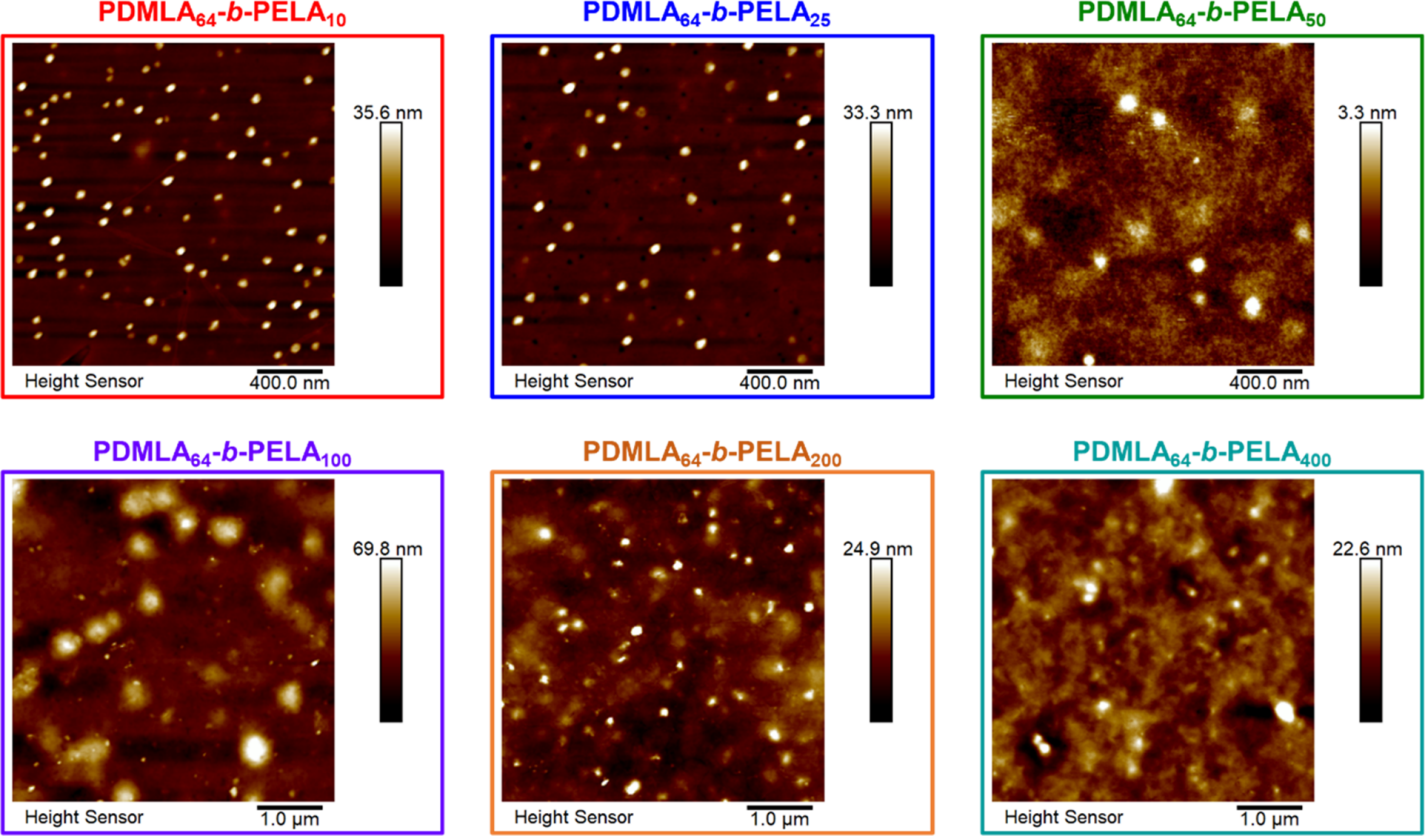
AFM height images of dried PDMLA_64_-*b*-PELA_*y*_ diblock copolymer nanoparticles, where *y* = 10–400, dried at 5 °C, synthesized by RAFT aqueous emulsion polymerization of ELA using either AIBA at 60 °C (*y* = 10, 25) or the redox pair AsAc/KPS at 30 °C (*y* = 50, 100, 200, and 400).

The low *T*_g_ of the PELA core-forming block enables the PDMLA_64_-*b*-PELA_*y*_ diblock copolymer nanoparticles to film form when dried at room temperature, resulting in an optically transparent film (Figure S14). As a result, characterization of the surface topography by atomic force microscopy (AFM) shows that a high degree of coalescence and particle identity is lost (see Figure S15). Aiming to reduce coalescence and visualize particle morphology, PDMLA_64_-*b*-PELA_*y*_ diblock copolymer nanoparticle dispersions were equilibrated overnight at ∼5 °C then dropped onto a glass substrate and allowed to dry at ∼5 °C before AFM topographical imaging; see Figure 4 and Table 4. While discrete spherical particles could be observed, the average diameters determined by image analysis of the AFM height images were typically larger than the *z*-average diameter measured using DLS by a factor of 2–3. This increase in average diameter, as observed by AFM, is the result of particle shrinking and flattening upon drying due to dehydration of the hydrophilic shells as well as particle deformation taking place while imaging at 21 °C. Moreover, the temperature at which the samples were prepared (∼5 °C) is very close to the *T*_g_ of the PELA core-forming blocks, *T*_g(1)_, thus some further particle deformation could be expected, particularly for the PDMLA_64_-*b*-PELA_200_ and PDMLA_64_-*b*-PELA_400_ with *T*_g(1)_ of 5.1 and 4.6, respectively. For example, the PDMLA_64_-*b*-PELA_50_ and PDMLA_64_-*b*-PELA_400_ particle heights were lower than expected, 2.9 and 9.7 nm, respectively, considering their *D*_*z*_ were 27 and 74 nm. However, for PDMLA_64_-*b*-PELA_100_ and PDMLA_64_-*b*-PELA_200_, the average heights were 27 and 25 nm, which were much closer to the recorded D_*z*_ of 30 and 42 nm, respectively, while average heights for PDMLA_64_-*b*-PELA_10_ and PDMLA_64_-*b*-PELA_25_ synthesized at 60 °C using AIBA were similar, were both 22 nm, and more significant than the corresponding *D*_*z*_ (11 and 17 nm), which suggests aggregation of some PDMLA_64_-*b*-PELA_*y*_ diblock copolymer nanoparticles upon drying.

**Table 4:** Average Diameter and Height of Spherical Objects Determined by Image Analysis of AFM Topography Images for Dried PDMLA_64_-*b*-PELA_*y*_ Diblock Copolymer Nanoparticles

target composition	average diameter (nm)	average height (nm)
PDMLA_64_-*b*-PELA_10_	49.3 ± 6.47	22.0 ± 2.1
PDMLA_64_-*b*-PELA_25_	57.8 ± 7.89	21.9 ± 3.5
PDMLA_64_-*b*-PELA_50_	76.0 ± 22.2	2.94 ± 0.8
PDMLA_64_-*b*-PELA_100_	90.5 ± 23.7	26.8 ± 14.6
PDMLA_64_-*b*-PELA_200_	129 ± 30.6	25.0 ± 6.9
PDMLA_64_-*b*-PELA_400_	157 ± 56.9	9.70 ± 2.9

### Temperature Response of PDMLA_*x*_ and PDMLA_64_-*b*-PELA_*y*_

As previously discussed, during the synthesis of PDMLA_64_-*b*-PELA_*y*_ diblock copolymer nanoparticles, it became evident that they were temperature responsive. Initially, to determine whether the PDMLA_*x*_ homopolymers exhibited any lower critical solution temperature (LCST) behavior, they were prepared at 5 mg mL^–1^ in deionized water (see Supporting Information for full method) to give transparent solutions and heated to observe any change in turbidity visually. Cloud points between 86 and 98 °C were observed for PDMLA_*x*_, where *x* was >50 (Table S2). The cloud point, *T*_c_, decreased with increasing PDMLA DP, demonstrating molecular weight-dependent LCST behavior. There was no cloud point observed for PDMLA_25_ and PDMLA_50_. Moreover, the PDMLA_64_-*b*-PELA_*y*_ diblock copolymer nanoparticle dispersions also increased turbidity with increasing temperature (Figure S16).

**Figure 5:**
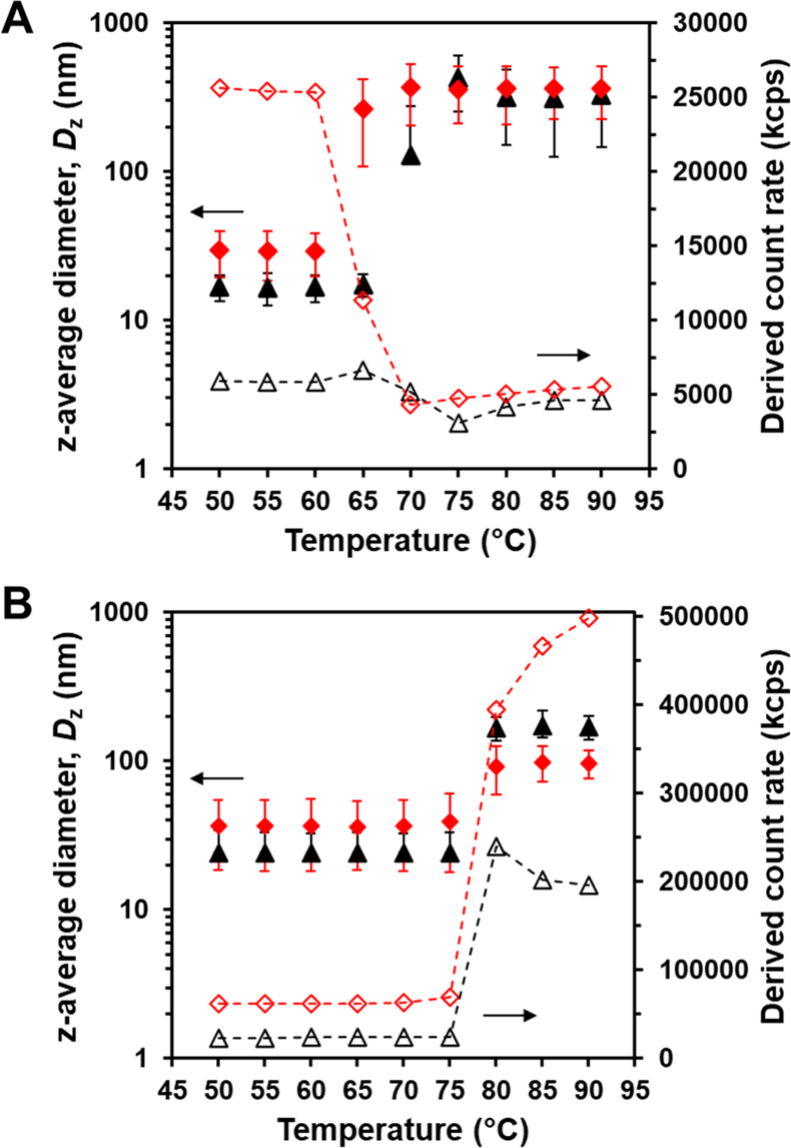
Z-average diameters (filled symbols) and derived count rates (open symbols) obtained from variable temperature DLS studies of PDMLA_64_-*b*-PELA_25_ (black triangles) and PDMLA_64_-*b*-PELA_50_ (red diamonds) synthesized using either: (A) AsAc/KPS or (B) AIBA as radical initiators. Error bars represent the standard deviation of the *z*-average diameter; dashed lines are provided for guidance only.

From visual observations, changes in turbidity were observed between 67 and 75 °C for PDMLA_64_-*b*-PELA_*y*_ diblock copolymer nanoparticle dispersions, where *y* = 10–100. Interestingly, the radical initiator used in the RAFT aqueous emulsion polymerization seemed to influence the temperature-responsive behavior. To further investigate this effect, variable temperature DLS experiments were conducted with PDMLA_64_-*b*-PELA_25_ and PDMLA_64_-*b*-PELA_50_, synthesized using either AIBA or AsAc/KPS; see Figure 5.

All samples showed a sudden increase in diameter (*D*_*z*_) with heating, suggesting either aggregation or a change in the particle morphology. For the PDMLA_64_-*b*-PELA_25_ and PDMLA_64_-*b*-PELA_50_ diblock copolymer nanoparticles synthesized at 30 °C using AsAc/KPS (Figure 5A), *D*_*z*_ increased from 17 and 29 nm at 50 °C, respectively, to over 300 nm when heated above 75 °C. PDMLA_64_-*b*-PELA_25_ (AsAc/KPS) increased in size significantly between 65 and 70 °C, while the increase in *D*_*z*_ for PDMLA_64_-*b*-PELA_50_ (AsAc/KPS) occurred between 60 and 65 °C. With increasing *D*_*z*_, the derived count rate decreased for both samples. This was unexpected, as an increase in the count rate would usually accompany an increase in size. However, this indicates that the diblock copolymer nanoparticles are unstable at higher temperatures. This was further corroborated by the concomitant increase in PDI observed for the particles upon heating from 50 to 90 °C, which also increased from 0.04 to 0.31 for PDMLA_64_-*b*-PELA_25_ (AsAc/KPS) and 0.12 to 0.15 for PDMLA_64_-*b*-PELA_50_ (AsAc/KPS) (data not shown). The larger objects could have sedimented in the DLS cuvette, leading to a reduction in the count rate detected by the DLS instrument. However, we were unable to confirm this visually, and these results warrant further investigation.

The PDMLA_64_-*b*-PELA_25_ and PDMLA_64_-*b*-PELA_50_ diblock copolymer nanoparticles synthesized at 60 °C using AIBA (Figure 5B) also increased in size with heating, from *D*_*z*_ of 24 and 37 nm at 50 °C to 169 and 92 nm at 80 °C, respectively. The size and count rate for both samples increased significantly when heating from 75 to 80 °C; for PDMLA_64_-*b*-PELA_25_ (AIBA), a subsequent decrease in count rate was observed with further heating, whereas the count rate recorded for PDMLA_64_-*b*-PELA_50_ (AIBA) continued to increase when heated above 80 °C. Furthermore, the PDI decreased with increasing temperature and *D*_*z*_, from a PDI of 0.16 to 0.03 and 0.26 to 0.05 for PDMLA_64_-*b*-PELA_25_ and PDMLA_64_-*b*-PELA_50_ (AIBA), respectively. This increase in count rate and decrease in PDI contrasts the behavior observed for the PDMLA_64_-*b*-PELA_25_ and PDMLA_64_-*b*-PELA_50_ (AsAc/KPS). It may suggest an increased colloidal stability at high temperatures for the diblock copolymer nanoparticles synthesized at 60 °C using AIBA. These initial investigations highlight differences in the temperature response of biobased diblock copolymer nanoparticles based on only a change in the radical initiator used during the synthesis and the reaction temperature used.

## Conclusions

Biobased PDMLA-*b*-PELA diblock copolymers based on lactic acid-derived green solvents have been synthesized by a combination of RAFT aqueous solution and emulsion polymerizations. First, the RAFT solution polymerization of DMLA was investigated using water and DMSO as “green” solvents using four different RAFT CTAs. Optimized reaction conditions were found using CECPA and water as the solvent, an ideal solvent for designing environmentally friendly syntheses. PDMLA homopolymers were prepared targeting DPs from 25 to 400. Kinetic investigations demonstrated that a high conversion (97%) could be achieved in as little as 1 h reaction time, and a linear increase in *M*_n_ was observed with increasing conversion. PDMLA was subsequently chain extended with ELA using RAFT aqueous emulsion polymerization, with either a thermal radical initiator (azo initiator) or a redox radical initiating system. Initial syntheses using ACVA with a reaction temperature of 70 °C, and AIBA at 60 °C, were not ideal due to unforeseen temperature-responsive behavior and excessive chain transfer leading to branching when targeting higher core-forming block DPs. Chain extension using the well-known redox pair ascorbic acid and KPS (AsAc/KPS) at 30 °C resulted in well-defined PDMLA_64_-*b*-PELA_*y*_ diblock copolymer nanoparticles, prepared at 10% w/w solids. Molecular weight dispersities, *Đ*, between 1.25 and 1.55 were obtained from SEC analyses, and the self-assembled diblock copolymer nanoparticles were characterized by DLS with diameters, *D*_*z*_, from 11 to 74 nm with low polydispersity indexes (PDI = 0.07–0.20). Subsequent investigation into their temperature-responsive properties found that PDMLA_*x*_, where *x* was >50, exhibited LCST behavior, while PDMLA_64_-*b*-PELA_*y*_ nanoparticles, where *y* was ≤100, showed an increase in the *z*-average diameter with heating.

This work demonstrates the first example of a biobased, stimuli-responsive diblock copolymer synthesized by RAFT aqueous emulsion polymerization. Here, the use of (i) abundantly bioavailable green solvents as raw materials, (ii) aqueous polymerizations, which are (iii) conducted at low temperature (30 °C) and achieve high monomer conversions within short reaction times (3 h), all adhere to the principles of green chemistry.^41^ Whereby (i) and (ii) address the use of *safer solvents* and (iii) contribute toward *design for energy efficiency*. Moreover, the film formation capabilities of these PDMLA_*x*_-*b*-PELA_*y*_ diblock copolymers suggest a potential application in coatings, while their intriguing temperature-responsive properties warrant further investigations and may open up to controlled release applications based on thermal triggers.

## Supporting Information Available

Experimental details including materials, characterization, cloud point determination protocol, and calculations to determine degree of polymerization (DP) by ^1^H NMR and chain transfer agent (CTA) efficiencies; chemical structures of the RAFT CTAs used in this study are provided, as are the ^1^H NMR spectra of the CTAs and corresponding PGMA_50_ homopolymers; FTIR analyses of the DMLA monomer and PDMLA_50_ homopolymer, obtained *M*_n_ versus target DP, and DSC analyses of PDMLA_*x*_ homopolymers are provided; PDMLA_*x*_-*b*-PELA_*y*_ diblock copolymer polymerization data is available, including monomer conversions obtained by ^1^H NMR, and analyses of the diblock copolymers by SEC, DSC, and DLS; moreover, representative ^1^H NMR spectra of PDMLA_64_ and PDMLA_64_-*b*-PELA_200_ are included; digital images of a dried film formed from PDMLA_64_-*b*-PELA_400_ and AFM images of diblock copolymer samples dried at ambient temperatures are provided; and cloud points recorded for PDMLA_*x*_ homopolymers are included with digital images exemplifying the transition (PDF)
