# Supplementary material for: Temperature-Responsive Lactic Acid-Based Nanoparticles by RAFT-Mediated Polymerization-Induced Self-Assembly in Water
Source: ACS Sustain Chem Eng. 2023 Jun 26;11(27):9979–88. doi: 10.1021/acssuschemeng.3c01112 (PMC10337250; doi:10.1021/acssuschemeng.3c01112)
Supplement: Supplementary file 1 — sc3c01112_si_001.pdf [file sc3c01112_si_001.pdf]

Supporting information

Temperature-responsive lactic acid-based nanoparticles by RAFT-mediated polymerization-induced self-assembly in water

Sarah E. Woods†, James David Tinkler†, Nabil Bensabeh‡, Marc Palà‡, Simon J. Martin†, Ignacio Martin-Fabiani†, Gerard Lligadas‡ and Fiona L. Hatton*†

† *Department of Materials, Loughborough University, Loughborough, LE11 3TU, UK*

‡ *Laboratory of Sustainable Polymers, Department of Analytical Chemistry and Organic Chemistry, University Rovira i Virgili, 43007 Tarragona, Spain*

Author information

*E-mail: f.hatton@lboro.ac.uk

Number of pages: 17 (including the cover page)
Number of Tables: 2
Number of Figures: 16

Contents

Experimental details

Materials……………………………………………………………………………3

Characterization…………………………………………………………………3

Cloud point determination using visual observation………………………………5

DP by NMR calculations……………………………………………………......6

CTA efficiency calculations……………………………………………………6

Figures and Tables

Figure S1…………………………………………………………………………7

Figure S2…………………………………………………………………………..7

Figure S3...………………………………………………………………………8

Figure S4...………………………………………………………………………8

Figure S5...………………………………………………………………………9

Figure S6...………………………………………………………………………9

Figure S7...……………………………………………………………………..10

Figure S8 ……………………………………………………………………..10

Table S1...………………………………………………………………………11

Figure S9...……………………………………………………………………..12

Figure S10...……………………………………………………………………12

Figure S11...……………………………………………………………………13

Figure S12...……………………………………………………………………13

Figure S13...……………………………………………………………………14

Figure S14...……………………………………………………………………15

Figure S15...……………………………………………………………………16

Table S2...………………………………………………………………………16

Figure S16...……………………………………………………………………17

Experimental details

Materials. *N,N*-dimethyl lactamide acrylate (DMLA) and ethyl lactate acrylate (ELA) were both synthesized using natural ethyl lactate (Merck, 98%) and D-(+)-*N,N*-dimethyl lactamide (DML) kindly donated by Corbion, Gorinchem (Netherlands).[16] 2,2′-azobis(2-methylpropionamidine) dihydrochloride (AIBA, 97%) was purchased from Fluorochem (Hadfield, UK). Deuterium oxide (D_2O, >99.9%) and dimethyl sulfoxide-d_6 (DMSO-d_6, >99.8 atom % D) were purchased from Apollo Scientific (Cheshire, UK). 4-((((2-carboxyethyl)thio)carbonothioyl)thio)-4-cyanopentanoic acid (CECPA, 95%) was purchased from Boron Molecular (Melbourne, Australia). 2-(2-carboxyethylsulfanyl-thiocarbonylsulfanyl)propionic acid (CPA, 95%), 2-(dodecylthiocarbonothioylthio)-2-methyl propionic acid (DDMAT, 98%), 4-cyano-4-(phenylcarbonothioylthio)pentanoic acid (CPADB), 4,4'-azobis (4-cyanovaleric acid) (ACVA, ≥98.0%), potassium persulfate (KPS, ≥99.0%), L-ascorbic acid (AsAc, ≥99%) and all other solvents were purchased from Merck (Missouri, USA). Snakeskin dialysis tubing with a molecular weight cut-off (MWCO) of 3.5 KDa was purchased from Thermofisher (Massachusetts, USA). Pre-wetted regenerated cellulose dialysis tubing with a MWCO of 1 KDa, and polystyrene (PS) macro-cuvettes were purchased from Fisher Scientific (Loughborough, UK).

Characterization. *Proton nuclear magnetic resonance (^1H NMR) spectroscopy***.** ^{1}H NMR spectra were recorded using either a JEOL ECS spectrometer at either 400 or 500 MHz using a 5 mm broadband probe or a Bruker AV3/400. Measurements were performed in D_2O, DMSO-d_6, or $CDCl_3$ using either 25 or 16 scans at 25 °C. The H_2O peak in PDMLA$_{64}$-*b*-PELA$_y$ was suppressed with 3 s DANTE presaturation pulse sequence.

Size exclusion chromatography (SEC). SEC was performed at 40 °C using an Agilent Technologies 1260 Infinity II Multi-Detector SEC/GPC system with a refractive index detector and two PLgel 5 μm MIXED-C columns. HPLC-grade chloroform ($CHCl_3$) containing 2% v/v triethylamine (TEA) and a toluene flow-rate marker at a flow rate of 1 mL min^{-1}. Calibration

was achieved using twelve Agilent EasiVial PS standards (M_p from 162 to 364,000 g mol^{-1}). PDMLA$_{64}$-*b*-PELA$_y$ diblock copolymer dispersions were dried in advance using a Thermo savant modulyo benchtop freeze drier.

Dynamic light scattering (DLS). A Malvern Zetasizer Pro DLS with an angle of detection of 173° (i.e., backscattering) analyzed PDMLA$_{64}$-*b*-PELA$_y$ nanoparticles using 5 mg mL^{-1} dispersions in deionized water. Isothermal tests were conducted at 25 °C. Variable-temperature tests (1.5 mL, 5 mg mL^{-1}, unadjusted pH 4.1 – 6.0) were equilibrated for 5 min at 50 °C, after which the temperature increased in 5 °C intervals up to 90 °C, with 3 min of equilibration before each measurement. The z-average diameter (D_z) and polydispersity index (PDI) were collected using automatic attenuation and measurement position. The derived count rate was determined by fixing the attenuator and measurement position.

Differential scanning calorimetry (DSC). Thermal analysis was conducted using 3 to 5 mg samples in a TA instruments Q200 DSC. A heat-cool-heat at 10 °C min^{-1} was used, from -20 to 160 °C for PDMLA$_x$ and -50 and 160 °C for PDMLA$_{64}$-*b*-PELA$_y$. The PDMLA$_{64}$-*b*-PELA$_y$ diblock copolymer dispersions were dried in an oven at 100 °C for 20 min prior to DSC analysis.

Fourier-transform infrared (FTIR) spectroscopy. FTIR was collected using a Shimadzu IR Tracer-100 FTIR spectrophotometer with a single reflection attenuated total reflectance (ATR) system using a 45° diamond positioned on the top plate.

Atomic force microscopy (AFM). AFM topography images of dried PDMLA$_{65}$-*b*-PELA$_y$ nanoparticle dispersions were collected at 21 °C with a Bruker BioScope Resolve using ScanAsyst imaging mode and silicon cantilevers (ScanAsyst-Air) at a typical spring constant of 0.4 N m^{-1} and tip radius of 2 nm. Images were obtained at three different positions per sample using scan sizes of 1-50 μm (depending on particle size). The 0.1% w/w dispersions in deionized water were equilibrated overnight in a refrigerator (5 °C). Using a pipette, 400 μL

of each was dispensed onto glass slides and dried in the refrigerator for a week. Samples prepared at ambient temperature were dried in a fume cupboard overnight.

Cloud point determination using visual observation. $PDMLA_x$ (unadjusted pH from 4.1 to 7.0) and $PDMLA_{64}$-*b*-$PELA_y$ (unadjusted pH from 4.3 to 6.3) were diluted in 5 mg mL^{-1}, placed into an oil bath at 70 and 50 °C, respectively, and equilibrated for 5 min. Afterward, the bath was increased in 5 °C increments with an equilibration of 3 min each time until either the liquid became cloudy or 100 °C was reached. For the copolymer dispersions, the temperature was either recorded when it changed from transparent or turbid or when a significant increase in turbidity was seen visually.

DP by ^{1}H NMR calculations

DP_{NMR} end-group analyses were conducted by integrating the signal b (3.55 – 3.77 ppm) peak of $-CH_2-$ protons in the $PDMLA_{50}$ spectrum (Figure S2) derived from the CECPA at the chain end (labeled with an arrow) (equation 1). This was then assigned to 2 protons and compared with the O-CH-R peak (5.31-5.64 ppm) in the repeating monomer unit, which corresponds to 1 proton.

$$DP_{NMR} = \frac{\int polymer\ peak}{no.\ protons} \Bigg/ \frac{\int end\ group\ peak}{no.\ protons} \quad (1)$$

For the other $PDMLA_{50}$ homopolymers, synthesized using CPA, DDMAT, and CPADB, a similar approach was used, using distinctive peaks for the CTA at the chain end and comparing them with the pendant monomer repeat unit. The RAFT agent CPA has a similar chemical structure to CECPA, with a peak at 3.55 – 3.77 ppm corresponding to a $-S-C\underline{H}_2-$. For DDMAT and CPADB, different end-group peaks were analyzed. For DDMAT, a CH_3 signal ($S(C{=}S)S-C(C\underline{H}_3)_2-COOH$) at 0.80-0.95 ppm with the integral corresponding to 3H, and for CPADB, the CH signal ($S(C{=}S)C{=}CH-C\underline{H}{=}CH-C\underline{H}{=}CH-C$) of the benzene ring at 7.57-7.45 ppm corresponding to 2H.

CTA efficiency

CTA efficiencies were calculated using equation 2, where DP_x is the targeted degree of polymerization.

$$CTA\ efficiency\ (\%) = \frac{DP_x}{DP_{NMR}}\ x\ 100 \quad (2)$$

Figures and Tables

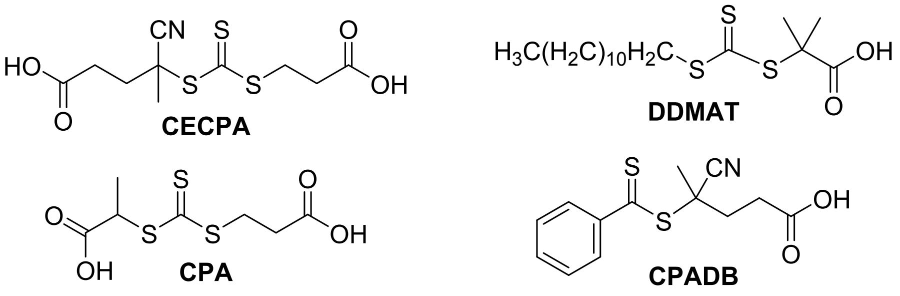

Figure S1. Chemical structures of the CTAs used for the RAFT aqueous solution polymerization of DMLA; 4-((((2-carboxyethyl)thio)carbonothioyl) thio)-4-cyanopentanoic acid (CECPA), 2-(2-carboxyethylsulfany-lthiocarbonylsulfanyl)propionic acid (CPA), 2-(dodecylthiocarbonothioylthio)-2-methyl propionic acid (DDMAT) and 4-cyano-4-(phenylcarbonothioylthio)pentanoic acid (CPADB).

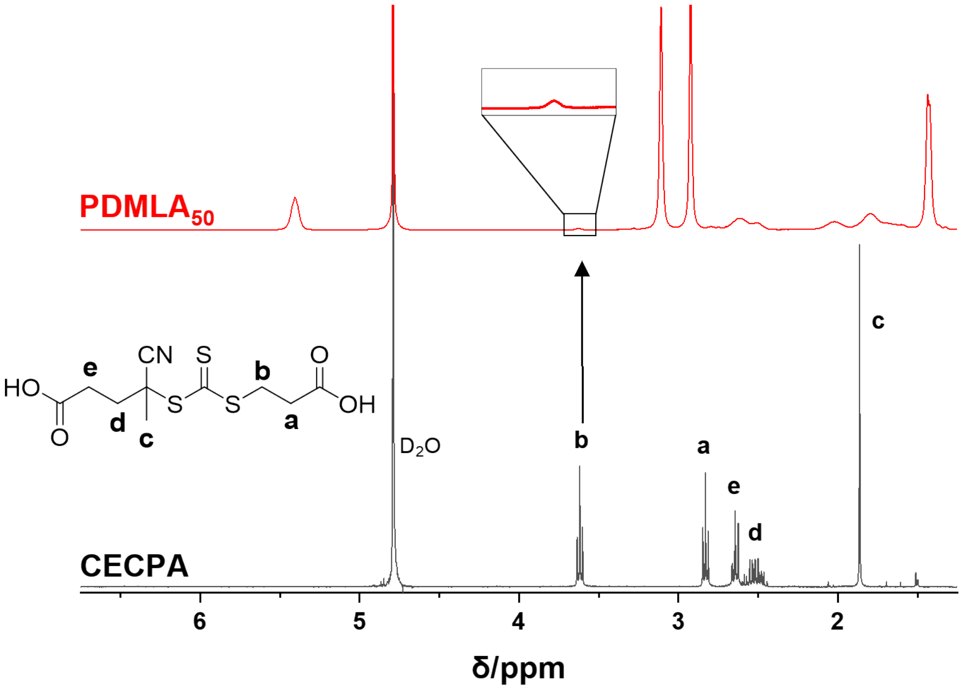

Figure S2. ^{1}H NMR spectra (400 MHz, D_2O) of CECPA and purified $PDMLA_{50}$, synthesized by RAFT aqueous solution polymerization with CECPA at 70 °C for 17 h. A DP_{NMR} of 52 was calculated using end-group analysis, suggesting a CECPA RAFT agent efficiency (DP_x/DP_{NMR}) of 96%.

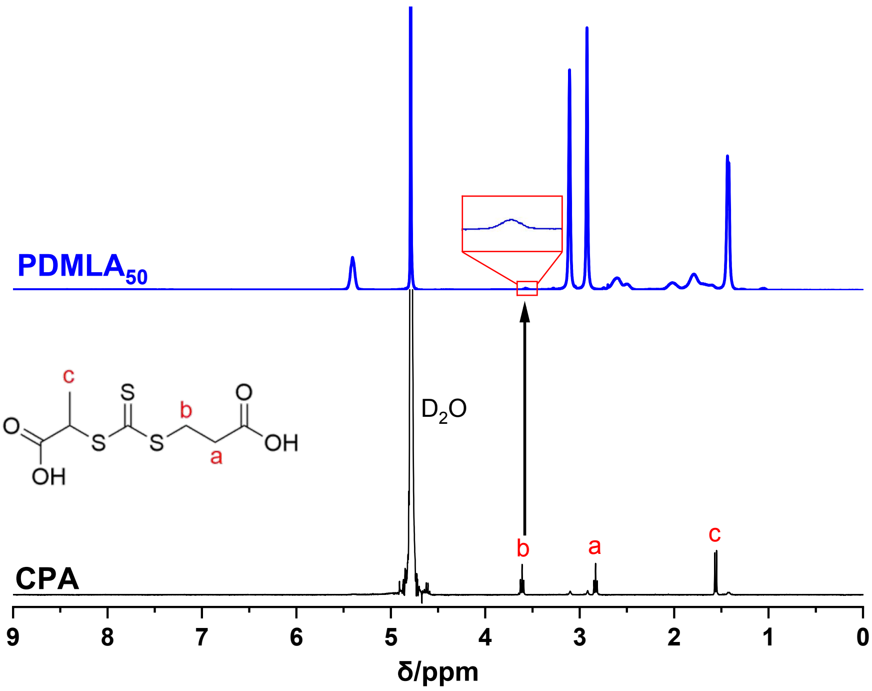

Figure S3. ^{1}H NMR spectra (400 MHz, D_2O) of CPA and purified $PDMLA_{50}$, synthesized by RAFT aqueous solution polymerization with CPA at 70 °C for 18 h. A DP_{NMR} of 65 was calculated using end-group analysis, suggesting a CPA RAFT agent efficiency (DP_x/DP_{NMR}) of 77%.

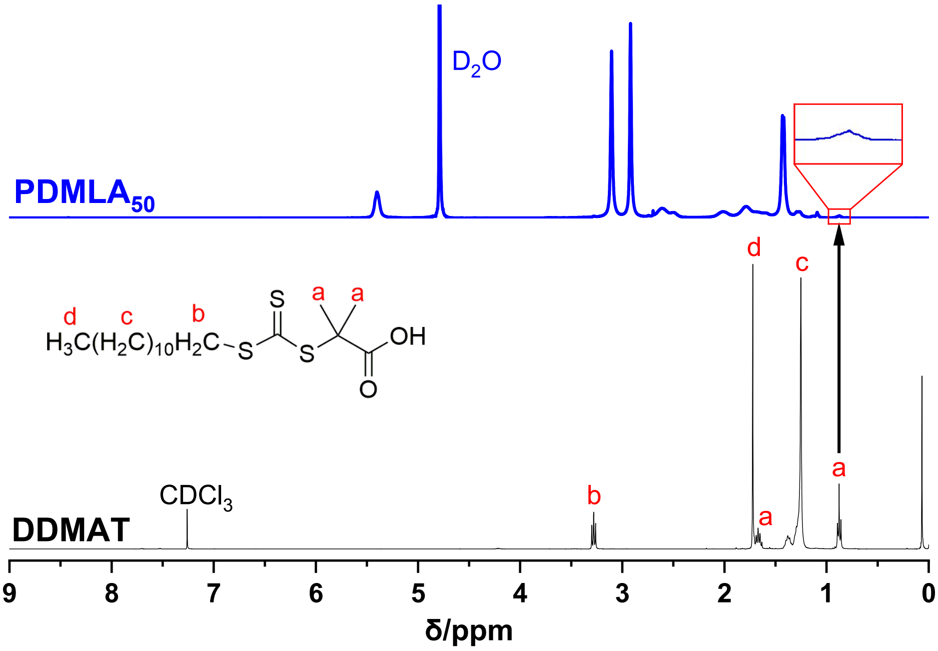

Figure S4. ^{1}H NMR spectra (400 MHz) of DDMAT ($CDCl_3$) and purified $PDMLA_{50}$ (D_2O), synthesized by RAFT solution polymerization in DMSO with DDMAT at 70 °C for 17 h. A DP_{NMR} of 57 was calculated using end-group analysis, suggesting a DDMAT RAFT agent efficiency (DP_x/DP_{NMR}) of 87%.

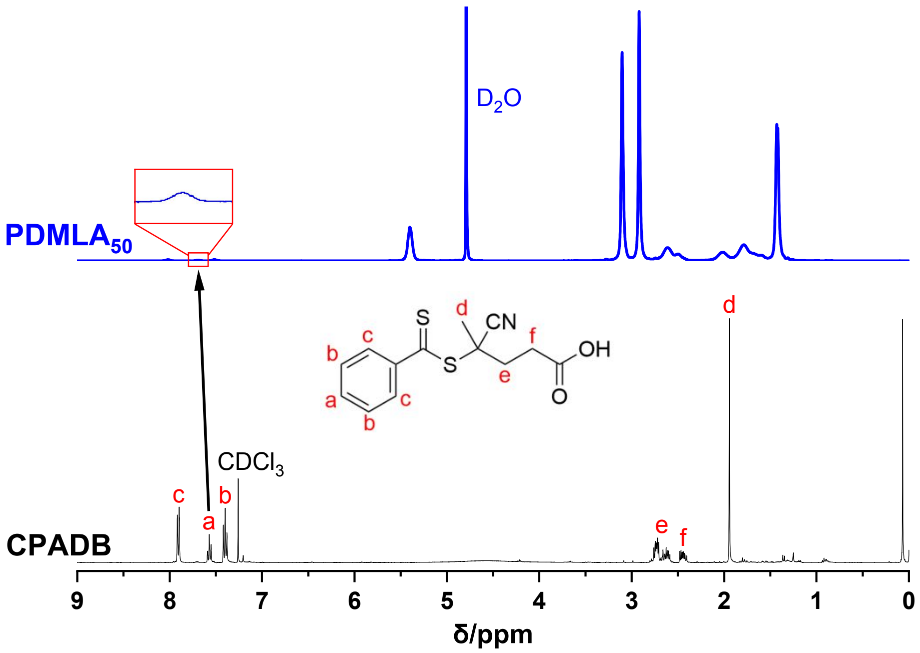

Figure S5. ^{1}H NMR spectra (400 MHz) of CPADB ($CDCl_3$) and purified PDMLA$_{50}$ (D_2O), synthesized by RAFT solution polymerization in DMSO with CPADB at 70 °C for 19 h. A DP_{NMR} of 78 was calculated using end-group analysis, suggesting a CPADB RAFT agent efficiency (DP_x/DP_{NMR}) of 61%.

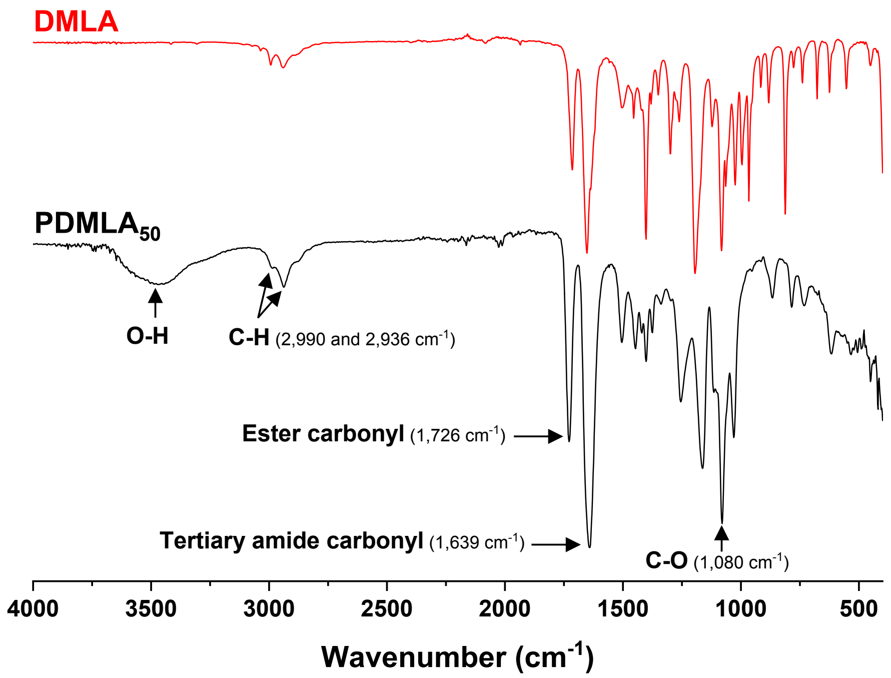

Figure S6. ATR-FTIR spectra of the DMLA monomer and PDMLA$_{50}$ homopolymer synthesized by RAFT aqueous solution polymerization using ACVA at 70 °C.

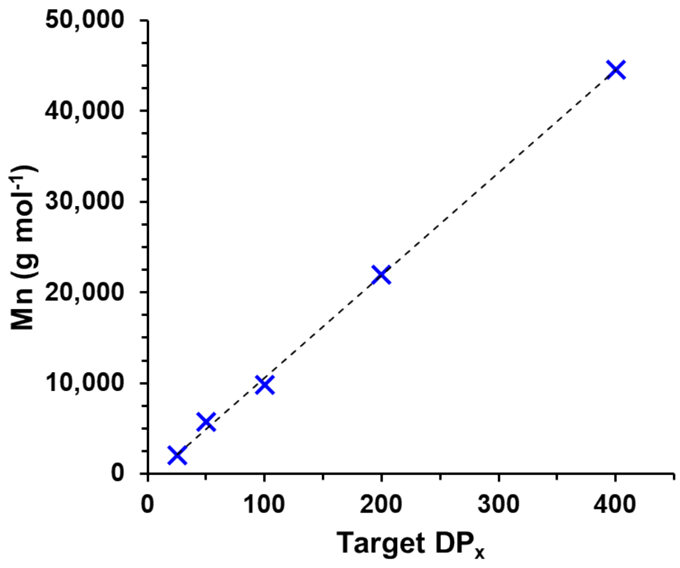

Figure S7. Number average molecular weight (M_n) versus target DP_x for a series of PDMLA homopolymers synthesized by RAFT aqueous solution polymerization of DMLA at 70 °C and CECPA/ACVA = 5, where x = 25, 50, 100, 200, and 400.

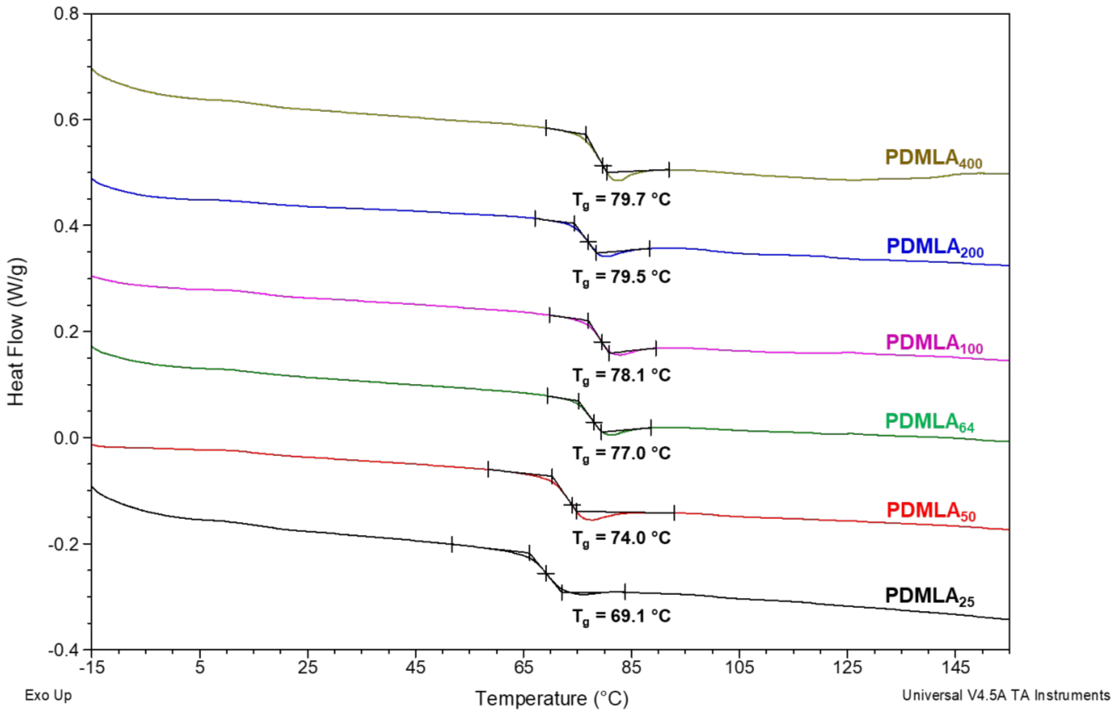

Figure S8. Second heating curves from differential scanning calorimetry (DSC) analyses of purified $PDMLA_x$ homopolymers synthesized by RAFT aqueous solution polymerization of DMLA at 70 °C and CECPA/ACVA = 5, where x = 25, 50, 64, 100, 200 and 400. The T_g is indicated on each thermogram.

Table S1. Monomer conversions, molecular weight, and dynamic light scattering data for the $PDMLA_{64}$-$PELA_y$ diblock copolymer nanoparticles synthesized using thermal azo-initiators ACVA and AIBA, at 70 and 60 °C, respectively. In all syntheses, the solids content was 10 and 20% w/w, macro-CTA/initiator = 5, with unadjusted solution pH from 3.2 – 4.0.

Target composition	Initiator used	Solids content (% w/w)	Conversion[a] (%)	M_n[b] (g mol^{-1})	$Đ$[b]	D_z[c] (nm)	PDI[c]
$PDMLA_{64}$-$PELA_{50}$	ACVA	10	96	14,800	1.40	69	0.28
$PDMLA_{64}$-$PELA_{10}$	AIBA	10	98	9,800	1.18	14	0.04
$PDMLA_{64}$-$PELA_{25}$	AIBA	10	>99	12,400	1.28	23	0.17
$PDMLA_{64}$-$PELA_{50}$	AIBA	10	99	16,000	1.32	37	0.37
$PDMLA_{64}$-$PELA_{10}$	AIBA	20	98	8,700	1.18	15	0.05
$PDMLA_{64}$-$PELA_{25}$	AIBA	20	99	10,900	1.22	19	0.08
$PDMLA_{64}$-$PELA_{50}$	AIBA	20	99	15,000	1.39	52	0.30
$PDMLA_{64}$-$PELA_{100}$	AIBA	10	96	14,400	3.31	145	0.21

[a] Determined by ^{1}H NMR analysis in d_6-DMSO.

[b] Determined by gel permeation chromatography analysis using $CHCl_3$ eluent containing 2% TEA and calibrated with a series of near-monodisperse poly(styrene) standards.

[c] Determined by dynamic light scattering at 5 mg mL^{-1}.

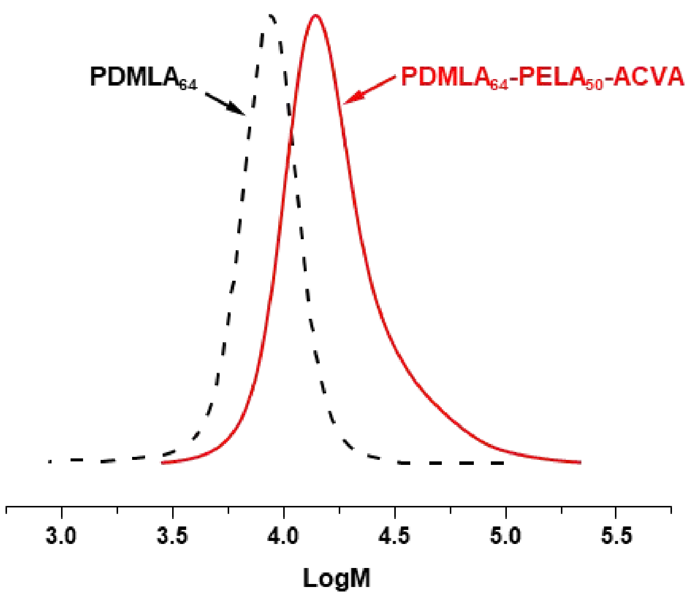

Figure S9. SEC chromatograms for the $PDMLA_{64}$ macro-CTA, synthesized by RAFT aqueous solution polymerization at 70 °C (black dashed line), and the $PDMLA_{64}$-b-$PELA_{50}$ diblock copolymer synthesized by RAFT aqueous emulsion polymerization of ELA using ACVA at 70 °C (solid red line).

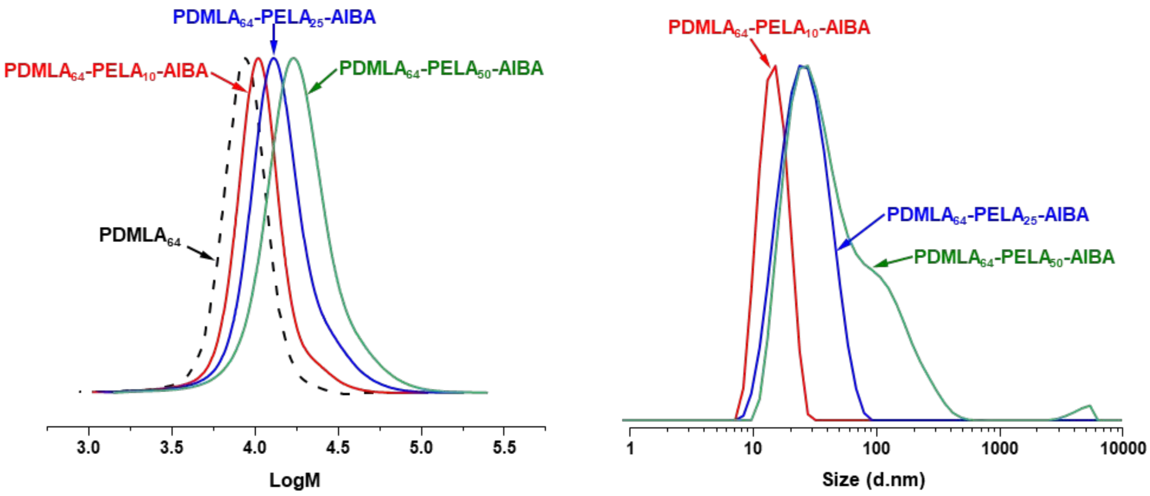

Figure S10. Results for $PDMLA_{64}$-*b*-$PELA_y$ diblock copolymers, where y = 10, 25, 50, synthesized by RAFT aqueous emulsion polymerization of ELA using the AIBA at 60 °C at 10% w/w solids content. A) Overlaid normalized SEC chromatograms and B) DLS size distribution by intensity curves for diblock copolymer nanoparticles.

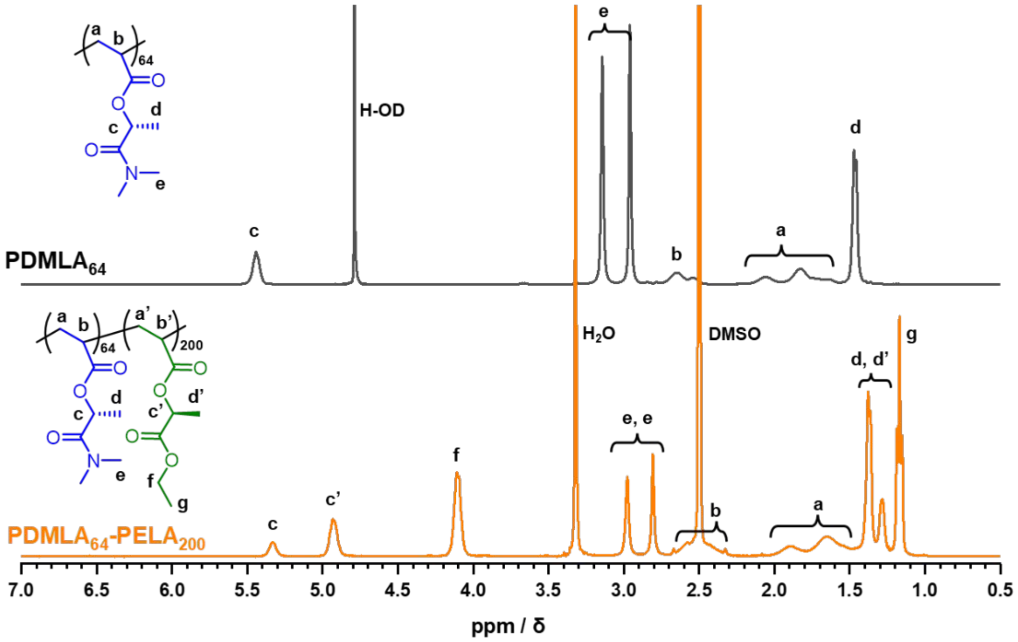

Figure S11. ^{1}H NMR spectra of $PDMLA_{64}$ macro-CTA (D_2O) synthesized by RAFT aqueous solution polymerization and $PDMLA_{64}$-*b*-$PELA_{200}$ diblock copolymer (DMSO-d_6) synthesized by RAFT aqueous emulsion polymerization of ELA, using AsAc/KPS at 30 °C.

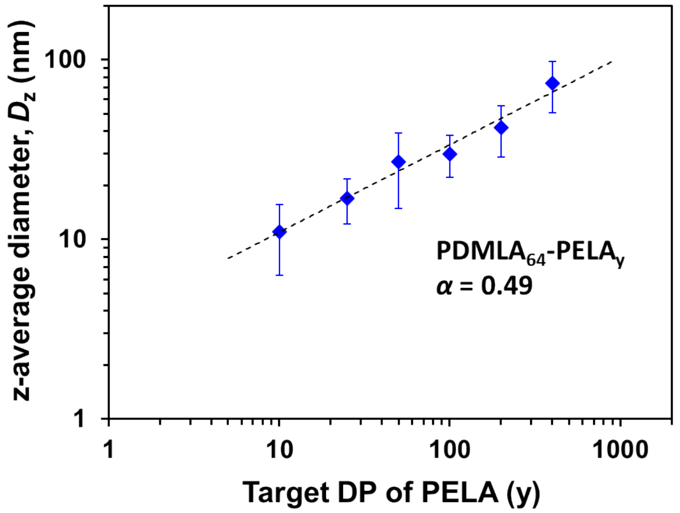

Figure S12. DLS data for $PDMLA_{64}$-*b*-$PELA_y$ diblock copolymer nanoparticles synthesized by RAFT aqueous emulsion polymerization of ELA at 30 °C using AsAc/KPS showing the relationship between z-average diameter and target PELA DP. The dashed line is the power law fit, α the scaling component, and error bars represent the standard deviation of the z-average diameter.

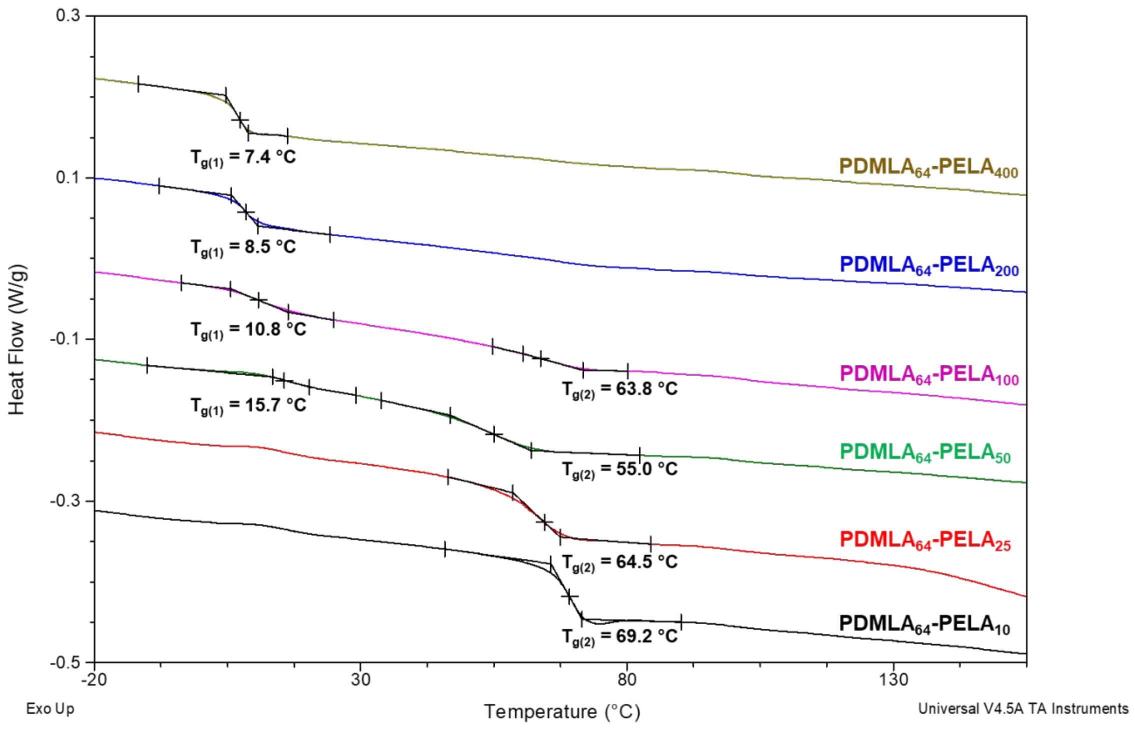

Figure S13. Second heating curves from differential scanning calorimetry (DSC) analyses of purified $PDMLA_{64}$-b-$PELA_y$ diblock copolymers synthesized by RAFT aqueous emulsion polymerization of ELA using AsAc/KPS at 30 °C where y = 10, 25, 50, 100, 200, and 400. The T_g is indicated on each thermogram.

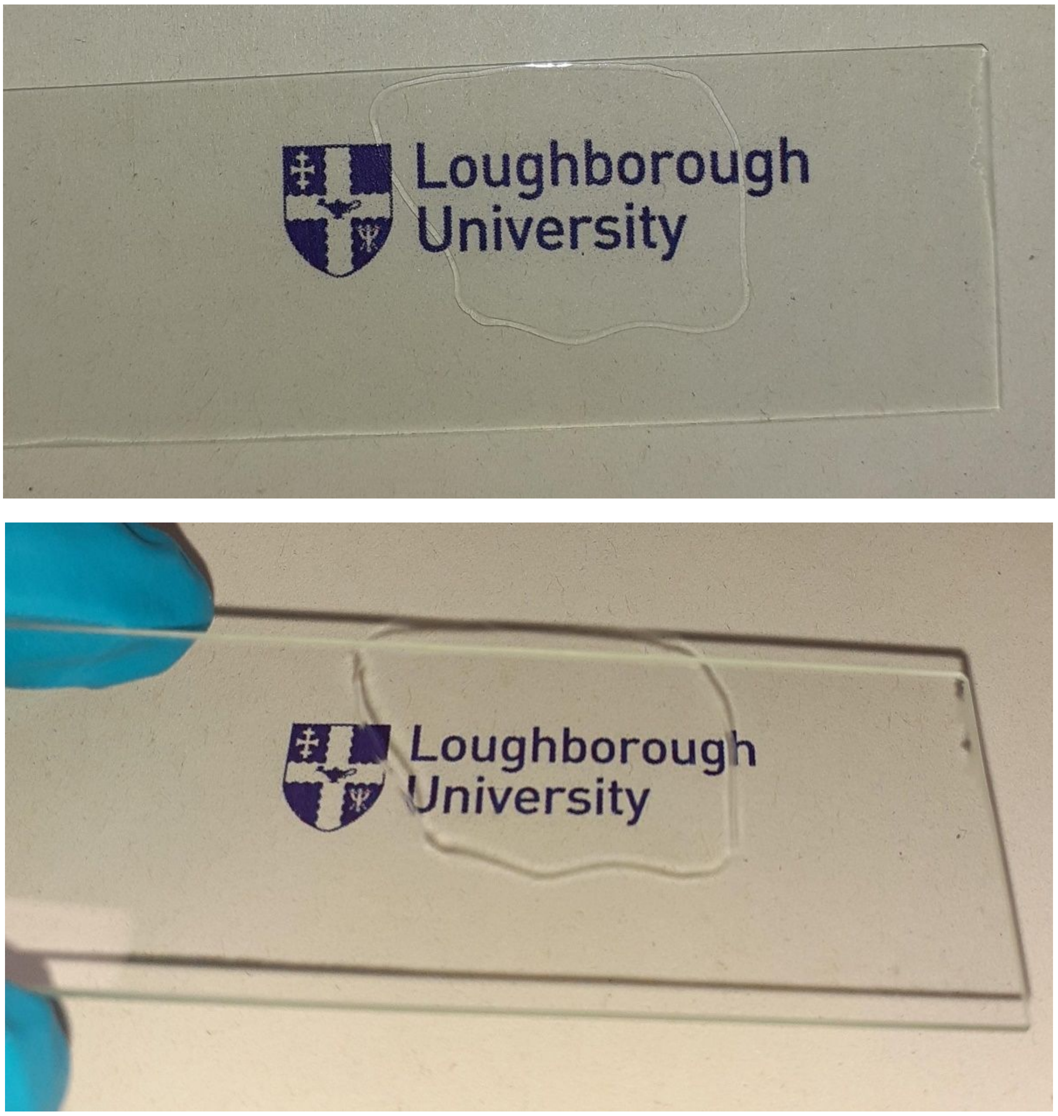

Figure S14. Digital photographs taken directly from above (top) and the side (bottom) of an optically transparent film formed from PDMLA$_{64}$-*b*-PELA$_{400}$ synthesized using AsAc/KPS at 30 °C, after 400 µL of a 0.1% w/w dispersion was cast onto a glass slide at room temperature and left to dry overnight. Permission has been granted by Loughborough University to reproduce the Loughborough University Logo in this Figure.

a

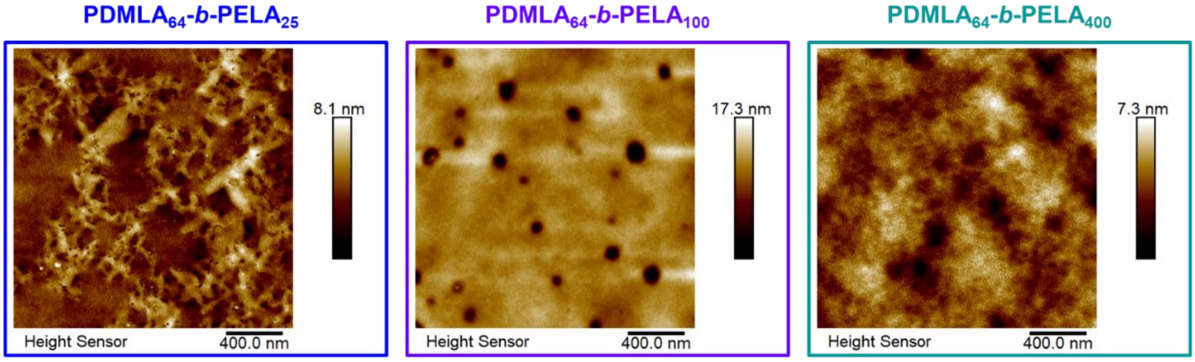

Figure S15. AFM height images of dried $PDMLA_{64}$-*b*-$PELA_y$ diblock copolymer nanoparticles, where y = 25 (AIBA), 100 (AsAc/KPS), 400 (AsAc/KPS), prepared at ambient temperature (~20 °C).

Table S2. Cloud points, T_c, determined from visual observations for $PDMLA_x$ homopolymer solutions (5 mg mL^{-1})

Target composition	T_c,[a] (°C)
$PDMLA_{25}$	-[b]
$PDMLA_{50}$	-[b]
$PDMLA_{64}$	98
$PDMLA_{100}$	98
$PDMLA_{200}$	90
$PDMLA_{400}$	86

[a] Determined by visual observation.

[b] No cloud point was observed.

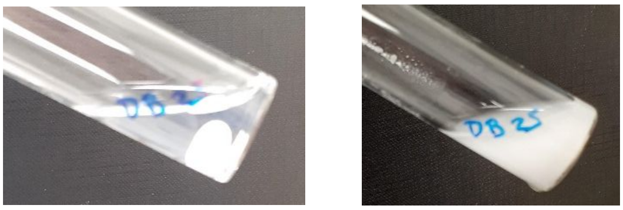

Figure S16. Digital photographs of $PDMLA_{64}$-$PELA_{25}$ synthesized using AIBA at ambient temperature (left) and above clouding point (right).
